# Implementation of Child Death Review in the Netherlands: results of a pilot study

**DOI:** 10.1186/s12913-016-1500-9

**Published:** 2016-07-08

**Authors:** Sandra Gijzen, Michaëla I. Hilhorst, Monique P. L’Hoir, Magda M. Boere-Boonekamp, Ariana Need

**Affiliations:** Department Health Technology and Services Research, University of Twente, Institute for Innovation and Governance Studies, PO Box 217, 7500 AE Enschede, The Netherlands; Department of Paediatrics, BovenIJ Hospital, PO Box 37610, 1030 BD Amsterdam, The Netherlands; Department of Forensic Medicine, Municipal Health Service region Utrecht, P.O. Box 51, Zeist, 3700 AB The Netherlands; TNO Child Health, P.O. Box 2215, 2301 CE Leiden, The Netherlands; Department Public Administration, University of Twente, Institute for Innovation and Governance Studies, PO Box 217, 7500 AE Enschede, The Netherlands

**Keywords:** SWOT, Child mortality, Child death review, Prevention, Quality of health care

## Abstract

**Background:**

Child mortality in the Netherlands declined gradually in the past decades. In total 1130 children and youth aged 0 to 19 years died in 2014 (i.e. 29.4 per 100,000 live births). A better understanding of the background and the circumstances surrounding the death of children as well as the manner and cause of death may lead to preventive measures. Child Death Review (CDR) is a method to systematically analyze child deaths by a multidisciplinary team to identify avoidable factors that may have contributed to the death and to give directions for prevention. CDR could be an addition to further reduce avoidable child deaths in the Netherlands. The purpose of this study is to explore the strengths, weaknesses, opportunities and threats (SWOT) of the pilot-implementation of CDR in a Dutch region. The results are translated in recommendations for future implementation of the CDR method in the Netherlands.

**Methods:**

Children who lived in the pilot region and died aged 29 days after birth until 2 years were, after parental consent, included for reviewing by a regional CDR team. Eighteen logs and seven transcribed records of CDR meetings concerning 6 deceased children were analyzed using Atlas ti. The SWOT framework was used to identify important themes.

**Results:**

The most important strengths identified were the expertise of and cooperation within the CDR team and the available materials. An important weakness was the poor cooperation of some professional groups. The fact that parents and professionals endorse the objective of CDR was an important opportunity. The lack of statutory basis was a threat.

**Conclusions:**

Many obstacles need to be taken away before large-scale implementation of CDR in the Netherlands becomes possible. The most important precondition for implementation is the acceptance among professionals and the statutory basis of the CDR method.

**Electronic supplementary material:**

The online version of this article (doi:10.1186/s12913-016-1500-9) contains supplementary material, which is available to authorized users.

## Background

Child mortality in the Netherlands has declined gradually in the past decades [3, 12]. In 2014, 1130 children in the age of 0 to 19 years died (mortality rate 29.4 per100,000 live births) [3]. In 8 out of 10 cases, the death was classified as due to a natural cause. Most children die in their first year, primarily due to conditions in the perinatal period and congenital abnormalities [3]. A better understanding of the background and the circumstances surrounding the death of a child as well as the manner and cause of death may lead to targeted preventive measures. In the Netherlands systematic analysis of child deaths only occurs in cases of Sudden Infant Death Syndrome (SIDS) by the National Cot Death Study Group [30] and in perinatal deaths by perinatal health care providers who participate in an obstetric collaboration [23]. Also, unexplained deaths in minors have been systematically examined in a Dutch pilot between October 2012 and January 2014. This so-called NODO-procedure (in Dutch *Nader Onderzoek DoodsOorzaak*) was regulated by law, and requested further investigation of the child’s death in order to clarify the primary cause of death [14, 15]. After its initial national pilot period, the Ministry of Health, Welfare and Sport concluded that further examination into the cause of death requested by the parents should be organized regionally in a less extensive procedure. In order to achieve this, organizations involved in child deaths are developing a multidisciplinary guideline right now that describes the procedure in case of unexplained death in minors [25].

A systematic analysis is not available for *all* child deaths in the Netherlands. In addition to the analysis of SIDS cases, perinatal deaths and unexplained death in minors, a standardized Child Death Review (CDR) could contribute to a further decline of avoidable child deaths in the Netherlands.

CDR is a method in which a multidisciplinary team systematically analyzes child deaths in order to identify avoidable factors that may have contributed to the death and that may give directions for prevention [5]. CDR has its origin in the United States of America (USA) where the first team started in the Los Angeles County in 1978. At first, the aim of CDR was to review suspicious child deaths in which abuse or neglect could have been a factor leading to the death. Gradually, CDR teams evolved in other states of America and some of them expanded their scope to reviewing *all* child deaths [8–10, 27]. Nowadays nearly half of the US states review child deaths from all causes [6]. In the late 1990’s, CDR was introduced in Canada and Australia [7] followed by New Zealand and the United Kingdom (UK) [1, 2, 10]. The implementation of CDR differs between these countries; not solely in the collection of data but also in legal foundation, focus, funding, family involvement and the location of the actual review [10, 33].

However different their implementation may be, studies have shown that CDR has the potential to identify avoidable factors in child deaths. For example, Child Fatality Review Teams in Arizona and Philadelphia (USA) concluded that 38 % and 37 % respectively of all deaths of children older than one month up to the age of 18 (and 21 respectively) years were considered preventable [21, 24]. In the UK it was concluded that 29 % of child deaths might be preventable [29]. In 20 % of the completed reviews in England in 2010 to 2011 modifiable factors in child deaths were identified [10]. These modifiable factors could be translated into effective intervention processes that might lead to a reduction in certain child deaths, like the safe sleep campaigns has resulted in a decrease in SIDS cases [4, 19, 22, 31] and the government traffic safety interventions that have reduced transport-related accidental deaths in children [12, 22].

To implement CDR in the Netherlands, support of organizations involved in child and family (health) care is required. Therefore, a bottom-up approach should be used to mobilize these organizations. This will ensure that CDR is effectively implemented, because in this way professionals involved are more motivated to adopt the method in their own practice [16].

In 2010, the authors of this paper conducted a feasibility study to examine which important parameters are needed to successfully implement the CDR. Three focus group sessions were held with professionals who are involved in a child’s death and one focus group with parents of a deceased child [13]. Based on the results of these focus groups we developed a strategy for implementation of CDR including a protocol that described the CDR procedure. Afterwards, a pilot implementation was started in the Eastern part of the Netherlands in January 2011 to determine to what extent the chosen implementation strategy was effective. This paper answers the following research question: which strengths, weaknesses, opportunities and threats in the pilot implementation of CDR can be identified and which recommendations can be made for future development of the CDR method in the Netherlands?

## Methods

### Study design

We used a qualitative, descriptive design to evaluate the pilot implementation of CDR in the Eastern part of the Netherlands. The SWOT framework, previously used as a tool for strategic management in the private sector [32], was used to identify Strengths and Weaknesses of an organization (i.e. internal environment) and Opportunities and Threats in the external environment (i.e. contextual factors as political, economic, social, technological, environmental and legal factors). The SWOT framework that is based on three pillars: stakeholder expectations, resources in the organization (i.e. people, means, finance and capabilities) and contextual factors, is suitable as a model for strategic analysis in the health care sector [32, 34]. In determining a strategy for further implementation, strengths and opportunities should be maximized and weaknesses and threats minimized [18].

### The Child Death Review protocol

The CDR procedure, described in a protocol [11], consisted of twelve steps that are outlined below. The CDR coordinator, who is also the researcher of this study (first author SG), fulfilled a secretarial role in this procedure.

#### Inclusion of cases

The death of a child living in the pilot region was notified by healthcare professionals who contacted the CDR coordinator by telephone or e-mail. In this contact they consulted the CDR coordinator about the best way to approach parents for reviewing their child’s death (first step). Next, the CDR coordinator asked a professional who has a confidential relationship with the parents to inform them about the CDR procedure in order to get informed consent for reviewing their child’s death (second step). To this end, specific written information material was made available to the parents. This professional notified the CDR coordinator (third step) as to whether the parents agreed to be approached by the CDR coordinator. When parents gave their permission, the CDR coordinator contacted the parents. In this contact parents were asked to give their consent for reviewing their child’s death by a CDR team (fourth step). The CDR team consisted of a chair, who is a forensic pediatrician, a general practitioner, a pediatrician, a preventive child health care physician, a forensic physician, a social worker and a psychotherapist. Then parents signed a consent form. After receiving this form, it was archived by the CDR coordinator (fifth step).

#### Intake

The CDR coordinator contacted all professionals who were involved before or around the time of death (sixth step). These professionals were asked to complete an intake form. This intake form was the same as used in the UK (i.e. agency report form; see Additional file 1) [27]. In a standard way, the general practitioner, the preventive child health care professional and the pediatrician, if involved, were approached. After receipt of all intake forms, the CDR coordinator wrote a chronological report with the assistance of the chair of the CDR team (seventh step). Then, the CDR coordinator anonymized all data (eighth step). Next the coordinator scheduled a CDR meeting (ninth step). To prepare for that meeting, the intake form and chronological report were sent to the CDR team members and chair.

#### CDR meeting

Before in the CDR meeting the review process started (tenth step) all CDR team members and the chair completed a confidentiality agreement. The CDR coordinator filled in the analysis *proforma* form. This form is used in the UK to analyze a child’s death [27] and was translated and adapted to the Dutch legislation and regulations (see Additional file 2). During the CDR meeting, factors intrinsic to the child, the family and environment, the parenting capacity and in relation to the service provision that may have contributed to the death, were identified. For all identified factors, the CDR team determined levels of influence. After the cause of death had been categorized, issues were identified and the CDR team formulated recommendations. The review ended with a follow-up plan for the family and possible actions (eleventh step). Finally, all data from reviewed cases were digitally archived in a secure environment (twelfth step) [17].

### Data collection

The target group of the CDR pilot project were all children living in a part of two (eastern) provinces of the Netherlands and who died aged between 29 days and 2 years in the period between January 2011 and December 2012. We chose this age category as child mortality in the Netherlands is the highest under the age of 2. Child deaths until 28 days after birth are reviewed in the Dutch perinatal audits [23], so these deceased children were not included in the CDR pilot project. Eighteen deceased children were reported. Signaling was done by eight pediatricians, five preventive child health care physicians, four forensic physicians and one Public Prosecutor. Of each of the eighteen deceased children, the CDR coordinator made a log. This log contains the name of the professional who notified the death and the date of reporting, names of other professionals involved, background information of the deceased child (age, gender, date of death, cause and place of death), and actions by the CDR coordinator to get parental consent. The process of obtaining parental consent is recorded by the CDR coordinator. Each log ended at the stage when parental consent became available or could not be obtained. In six out of eighteen deceased children, the parents gave their consent for reviewing the death of their child. Hence these six cases were included in the study. They were reviewed in seven CDR meetings. The review of each deceased child was scheduled in a one-hour meeting. Because the CDR team had to get used to the CDR method, the review of the first deceased child took two meetings. Each CDR meeting was audiotaped with consent of all CDR team members. The first author transcribed the recordings verbally.

In the reviews of the cases, factors have been identified that may have contributed to an increased vulnerability, ill health or even death. The CDR team has also identified factors that provide a complete and sufficient explanation for the death in the domains child’s needs, family and environment and service provision (see Table 1).

**Table 1:** Number of cases in which factors, arranged per domain, were identified that may have contributed to vulnerability, ill-health or death or that provide a complete or sufficient explanation for the death, based on the review of the 6 cases

Domains	Number of cases in which the factor was identified
Child's needs	
Acute/sudden onset illness	4
Chronic long term illness	
Epilepsy	1
Other chronic illness	4
Disability of impairment	
Motor impairment	2
Other disability or impairment	3
Family and environment	
Condition	
Emotional/behavioural/mental health condition in a parent or caregiver	1
Smoking by the parent/caregiver in household or during pregnancy	1
Parenting capacity	0
Service provision	2

Eighteen logs and seven transcribed records of the CDR meetings concerning six deceased children were used for analysis after the CDR coordinator had anonymized the data.

### Data analysis

The logs and transcripts were analyzed according to the SWOT-framework using the software program Atlas.ti [20]. We defined ‘strength’ as any resources in the CDR team that inspired the team to be effective. Any resources in the CDR team that hindered progress of the CDR team were considered to be a ‘weakness’. ‘Opportunity’ was defined as any contextual factor that promoted the execution of tasks by the individual professionals in the CDR team or the CDR team as organizational unit. Conversely, ‘threat’ was defined as any contextual factor that could act as a barrier to the execution of tasks by the professionals in the CDR team or the CDR team as organizational unit.

The first author analyzed the documents first and coded relevant text fragments according to a coding scheme. In this coding scheme the CDR team was seen as the organization (i.e. internal environment) operating within the broader organizational system in the Netherlands (i.e. external environment). Every resource or contextual factor that could be interpreted as respectively a strength or weakness or opportunity or threat was provided by a code. Stakeholders who play an important role for the optimal functioning of the organization were listed in the coding scheme and were provided with a code as well. Only text fragments in relation to the external environment were combined with codes of the stakeholders. In case the role of the stakeholder was mentioned in the text fragment, the associated code was added to the code of the contextual factor concerned. Next, the fourth author (MB) independently coded the relevant text fragments in the same way. Both authors compared the codes and the corresponding text fragments. Differences were discussed until consensus was reached.

## Results

### Strengths

Strengths could be identified in people, means and finance that inspired the development of the CDR team as shown in Table 2. The CDR coordinator provided additional information about the aim and procedure of CDR to professionals and parents. The forensic and pediatric expertise of the chair proved to be very valuable in the preparation of the CDR meetings as well as during the reviews, in which she approached each case from a broad view. Furthermore, it turned out that the CDR team members perceived the multidisciplinary approach as valuable; they complemented each other in a positive way. Due to the composition of the team they also called each other’s attention to stick to the facts and not interpret when analyzing a child’s death. They also were committed and cooperated as a team in order to improve the CDR procedure.

**Table 2:** Overview of the strengths, weaknesses, opportunities and threats identified in the child death review pilot study

Strengths	Weaknesses
People • CDR coordinator - contributed to the inclusion of cases • Chair - forensic and pediatric expertise - supported in writing the chronological report • Team - multidisciplinary approach - made proposals and recommendations directed at the intake and analysis of cases - reached consensus to refine the CDR procedure Means • Materials to inform parents and professionals (leaflet for parents, consent form, SERRAFIM website) • Materials to review a case (DVD ‘Why Jason died’, CDR protocol, confidentiality agreement, document with rules for an efficient meeting, intake and analysis form, recording equipment) Finance • Fee for chair and CDR team members • Reimbursement of travel expenses	People • CDR coordinator - could not complete the process to get parental consent in 2 cases in time - failed once to send the documents for the CDR meeting in time - has insufficient expertise to select relevant information from the medical record of the deceased child • Chair and CDR team members - voluntariness of participation • CDR team - limited experience Means • Lack of essential information from professionals and parents • Illogical ranking of items on the intake and analysis forms • Lack of a clear description how to define the primary and secondary cause of death and the way a death should be classified Finance • Insufficient financial resources for the CDR coordinator
Opportunities	Threats
Political factors • Added value to the legally prescribed NODO-procedure • National attention of the topic ‘child death’ • Providing a source of information for professionals, parents and others Social factors • The objectives of the CDR promoted participation Environmental factors • Collaboration with the National Cot Death Study Group • Reduction of the effort for parents in providing information • Highlighting positive experiences • Presenting at conferences or meetings and publishing in national magazines • Making use of experiences with conducting reviews nationally and in the UK Legal factors • Cooperation of the Public Prosecutor • Signed consent form to obtain information	Political factors • The influence of the NODO-procedure on the inclusion of cases Social factors • Personal reasons of parents and professionals for not participating Environmental factors • Influence of the Dutch Healthcare Authority on the participation of health care professionals in the CDR team Legal factors • Lack of statutory basis

The written materials and the special website, called SERRAFIM (Systematic Evaluation with Risk analysis and Review of Adverse Factors in Infant and child Mortality) [26], were supportive in informing parents and professionals about the CDR procedure and obtaining parental consent. During the meetings the materials available to review a child’s death seemed to be helpful to set the parameters in which the CDR team functions and to structure the information and review process.

With regard to the financial resources, strengths were identified in the fact that the CDR team members and chair were rewarded for their effort and travel expenses were reimbursed.

### Weaknesses

Weaknesses could be identified in people, means, finance and capabilities that hindered the progress of the CDR team as presented in Table 2. In the process of obtaining parental consent, the intake and the preparation of the CDR meeting the CDR coordinator was not always able to act according to the determined procedure. Another weakness was identified in the fact that the chair and CDR team members participated alongside their own practice or had other obligations, which affected the continuity in the team. Due to personal reasons the chair needed to be replaced by a team member with limited experience. Other engagements hindered the preparation of some cases by the chair and the attendance of some of the team members. Furthermore, the CDR team needed time to gain enough experience with the CDR method to be able to review the cases efficiently. The view on service provision differed between the members with their different backgrounds. This limited the number of cases that could be discussed in one meeting.

Due to lack of essential information from professionals involved and from the parents in one case, not every death of a child could be analyzed properly. First of all, professionals did not always provide the information needed to understand the mechanism of death. Second, in some cases of infant death it appeared that also the gynecologist and midwife had to be approached by the CDR coordinator for information. However, as perinatal deaths were excluded in our project, approaching the gynecologist and midwife was not a standard procedure in our protocol. Therefore, essential information about the period during pregnancy and labor and after birth could not always be obtained. Third, information about the primary and secondary cause of death written down on the medical death certificate was always lacking and could not be provided to the CDR coordinator. It is not a custom to keep a copy of this certificate in the deceased child’s medical file. Furthermore, it is not possible to request the individual medical death certificates from Statistics Netherlands. Next to the lack of essential information weaknesses were also found in the intake and analysis forms with regard to certain items that were not clear in terms of ranking or description.

The lack of sufficient financial resources hindered the CDR coordinator to invest sufficient time and thereby to fulfill her function optimally. The lack of time prohibited the CDR coordinator in keeping the inventory of child deaths in the pilot region up to date; this proved to be rather labor-intensive as professionals did not contact the CDR coordinator themselves to notify the death of a child.

### Opportunities

Contextual factors that promoted the execution of tasks by the CDR team could be found in political, social, environmental and legal factors as shown in Table 2. As a result of the experiences in the pilot study, one of the CDR team members indicated that CDR might be an addition to the NODO-procedure in cases of an unexpected child death. Furthermore, CDR might have another status when there is more attention to it nationally and when its aim is expanding in terms of providing a source of information for professionals, parents and others who are interested.

It turned out that parents who gave their consent and professionals who are involved in a child’s death endorsed the objective of CDR that promoted them to participate. In most cases, the pediatrician next to the preventive child health care professional contributed in approaching parents to inform them about CDR. After parental consent the professionals who are involved, such as the pediatrician, general practitioner, preventive child health care professional, forensic physician and attorney, have contributed in providing information to the CDR coordinator.

In the pilot study the collaboration with the National Cot Death Study Group turned out to be valuable with regard to offering support to parents. This Study Group also offered to provide information after parental consent in two cases of sudden infant death that were notified by professionals. Increasing the participation of parents and professionals and the awareness of CDR were other opportunities identified. Next to this, exchanging experiences with conducting reviews was also recognized as an opportunity. It turned out that the same learning curve was present during the implementation of the perinatal audits in the Netherlands to get familiar with the method. With regard to legal factors the participation of the Public Prosecutor and the pediatricians in the provision of information only in the presence of a signed consent form was also seen as an opportunity.

### Threats

Contextual factors acting as a barrier to the execution of tasks by the CDR team could be identified in political, social, environmental and legal factors as presented in Table 2. The NODO-procedure was identified as a threat for the implementation of CDR in three ways. First, it proved that professionals became confused to which procedure they needed to act when a child had died. Second, the poor implementation of the NODO-procedure contributed to a negative attitude of pediatricians to participate in CDR. Third, in case a child’s death was included in the NODO-procedure and parents gave their consent for CDR it was likely to be difficult to obtain information gathered in the NODO-procedure. This information should be gathered again in order to review the death.

The way a verdict of the Dutch Healthcare Authority could influence the participation of a CDR team member was also identified as a threat. Furthermore, the lack of statutory basis limited the benefits of CDR.

The fact that parents and some professionals, such as a pediatrician and preventive child health care physician or nurse, did not want to participate in CDR limited the number of cases to be reviewed. In five out of twelve cases that could not be reviewed, parents gave a reason for not participating. Some parents indicated that they did not want to talk about their deceased child or wanted to be left alone. Others, going through a difficult time, did not have the energy to focus on other things besides themselves. One parent did not want to further let examine the death of her child. In one of the remaining seven cases the attending physician indicated that the cause of death was clear and the case would therefore not be suitable for CDR. In another case the preventive child health care professional perceived that CDR would not have an added value compared to the review conducted by the National Cot Death Study Group. In the same case communication problems between parents and the professional was reported as a possible reason for the professional not to participate in CDR. Another reason was the extra burden for parents when information gathered during the NODO-procedure would not be available for CDR, as indicated by a professional in another case.

## Discussion

In this study we examined the strengths, weaknesses, opportunities and threats in the implementation of CDR in a pilot region in the Eastern part of the Netherlands. We used the SWOT framework to analyze the logs and transcribed records of the CDR meetings. The findings provided recommendations for future implementation of CDR not only in the Netherlands, but also in other countries that consider establishing CDR teams.

Strengths are identified in the contribution of the CDR coordinator, the expertise of the second chair and team members, the available materials and the multidisciplinary approach. Similarly to what has been concluded in other studies, sufficient experience and a multidisciplinary team that conducts reviews in an atmosphere of trust is needed to be effective [10, 28]. It is also known that committed team members are necessary to operate effectively as concluded in a study where teams just started to evolve in England [30]. Next to this, sufficient resources as funding of administrative staff and professionals’ time are required to function optimally [28]. It turned out that the available financial resources were sufficient for the chair and CDR team members, which is another strength.

Three identified weaknesses were: 1. the insufficient time and financial resources for the CDR coordinator to fulfill her function optimally, 2. other engagements of the chair and team members which affected the continuity within the team and, and 3. the fact that the CDR team could not always analyze a child’s death properly because essential information, e.g. primary and secondary causes of death, from professionals was lacking. Although the available materials were supportive for the CDR team to be able to review a child’s death, during the pilot some of the forms were adjusted to improve their use.

The benefits of CDR that promoted parents and professionals to participate and might be a valuable addition to the analysis of unexplained death in minors in the so-called NODO-procedure, were identified as an opportunity. Other opportunities were identified in reducing the effort for parents and highlighting the positive experiences to increase the participation of parents and professionals.

Confusion among professionals caused by the NODO-procedure that had just been introduced, the lack of statutory basis and personal reasons parents and professionals could have for rejecting participating in CDR, proved to be important threats for the notification of child deaths to the CDR coordinator. Together with the time constraints the CDR coordinator was facing, eighteen of the estimated total of 38 child deaths were captured in the pilot region during the study period. The insufficient participation of professionals could also be explained by the fact that they might have some degree of anxiety to provide information.

### Strengths and limitations of this study

One major strength of this study is the collaboration with (inter)national experts in the field of reviewing child deaths, which improved the quality of the review process. During the pilot study experiences were exchanged and uncertainties with regard to the CDR method were discussed in order to review a child’s death in the same way as in the UK. This provided the opportunity to compare the implementation of the procedure between the pilot region in the Netherlands and the UK.

The SWOT framework proved to be a suitable tool for analysis of the implementation of CDR, because this framework provided us specific points for future implementation. As we considered political, environmental and legal factors not only on a local but also on a national level in this study, the results might be used in other parts of the Netherlands and in other countries that consider to implement CDR. However, the framework should be used in a larger and representative group of deceased children, to be able to conclude whether this CDR protocol is the most suitable protocol to conduct CDR’s.

The fact that the chair and CDR team members were highly motivated to make recommendations in order to improve the CDR method and to make proposals in order to put CDR on a national agenda was another strength.

The researcher (SG) fulfilled the secretarial function of CDR coordinator, but did not participate in the assessment of the cases. To prevent the risk of bias, the researcher and a second coder (MB) analyzed the logs and transcribed records. The fact that the logs with data on characteristics of the deceased child and with actions set out by the CDR coordinator to get parental consent were filled out without the use of a predetermined structure was another limitation. If we had collected these data in a more structural way, the logs could have provided us with more specific information about the deceased children and the reasons why professionals and parents did not participate in the study. Finally, the relatively small number of the logs and transcribed records has implications for the conclusions of this study, which need to be drawn carefully. As long as there is no legal obligation to review child deaths, there is the risk of selection bias. Furthermore, CDR is not generally accepted among professionals involved in child deaths. If there had been a higher level of acceptance, we could have included more cases and this study probably would have provided us more detailed and valuable information.

## Conclusions

This study must be seen as a first introduction and exploration of application of the methodology of CDR in the Netherlands. The multidisciplinary approach and the endorsement of the CDR objectives by parents and professionals turned out to be the most important strengths and opportunities in the implementation of CDR. The insufficient time and finances, the existence of other Child Death Review processes and the lack of statutory basis are identified as important weaknesses and threats. These obstacles need to be taken away before large-scale implementation of CDR becomes possible. The most important precondition for implementation is the acceptance among professionals and the statutory basis of the CDR method. Acceptance among professionals can be enlarged by the incorporation in professional standards, preferably supported by the management of healthcare organizations. Next to this, it should be considered how to better integrate Child Death Review processes that have partly different and partly overlapping objectives and target groups. More research is needed to find out which Child Death Review process is feasible to use for certain child deaths.

## Abbreviations

CDR, Child Death Review; NODO, in Dutch: Nader Onderzoek Doodsoorzaak; SERRAFIM, Systematic Evaluation with Risk analysis and Review of Adverse Factors in Infant and child Mortality; SIDS, sudden infant death syndrome; SWOT, Strengths, Weaknesses, Opportunities, Threats

## Additional files

Additional file 1: Form B - Agency Report Form. (DOC 334 kb) Additional file 2: Form C - Analysis Proforma. (ODT 32 kb)
