# Supplementary material for: Implementation of Child Death Review in the Netherlands: results of a pilot study
Source: BMC Health Serv Res. 2016 Jul 8;16:235. doi: 10.1186/s12913-016-1500-9 (PMC4938929; doi:10.1186/s12913-016-1500-9)
Supplement: Additional file 1: — Form B - Agency Report Form. (DOC 334 kb) [file 12913_2016_1500_MOESM1_ESM.doc]

**Form B - Agency Report Form**

This form to be returned to CDOP Manager at: Email:

Address:       Fax:

**The information on these forms and the security for transferring it should be clarified and agreed with your local Caldicott guardian.**

**Please complete this form based on the information you have and return it quickly to the CDOP manager. If in doubt about what information to provide, please discuss with your manager.**

**Completing the form:** The form is sent out to all agencies involved with a child and family. As such you are not expected to complete all of the form. **You are asked to complete only those sections and questions on which you hold information**. Some information is collected in tick box or yes/no format to allow collation and comparison of data, but in each section there is space for more narrative/qualitative information which will help the CDOP to more fully understand the nature of each child’s death. If you do not have information for any particular item, please either circle or tick NK (Not Known) or NA (Not Applicable) or leave the item blank. It is preferable to circle or tick not known as this indicates to the CDOP that you have considered the question but have no information.

The form consists of six sections, A to F, along with supplementary forms B2 – B12 to be completed where appropriate according to the type of death. **Please note:** **If the death concerns the death of a neonate please complete form B2 first.**

**Purpose**: Form B is designed to gather information about each child’s death. Its primary purpose is to enable the local CDOP to review all children’s deaths in their area in order to understand patterns and factors contributing to children’s deaths and ultimately to take steps to prevent future child deaths.

**Confidentiality:** The information requested on this form will be used for the purposes of child death review as outlined in chapter 7 of Working Together. All bereaved parents are informed of these processes. The nature of the information collected means it is likely that some of the information is personal/sensitive data and therefore CDOPs should be mindful of their obligations under the Data Protection Act (DPA) 1998 when processing that information. All cases will be anonymised prior to discussion by the CDOP. All information gathered will be stored securely and only anonymised data will be collated at a regional or national level.

**This page may be removed for the purposes of anonymisation prior to discussion at the CDOP**

**A: Identifying and Reporting Details**

| Full name of child |  | | | Date of birth | /    / |
| --- | --- | --- | --- | --- | --- |
| NHS No. |  | | | Date of death | /    / |
| Gender | Male |  |  | | |
|  | Female |  |
| Address  (including postcode if known) |  | | | | |

**Agency Report Provided by**

| Agency |  | | Name | |  |
| --- | --- | --- | --- | --- | --- |
| Address |  | | | | |
| Postcode |  | | | | |
| Tel No |  | Email | |  | |

**B: Summary of Case and Circumstances leading to the death**

*This section provides information on the nature and manner of the child’s death. Please complete any information which you hold on the case.*

**The ‘Details of the Death’ section is to be completed by the treating doctor involved with the child at the time of death – other professionals can complete this section if they have the information.**

| **Details of the Death** | | | |
| --- | --- | --- | --- |
| What is your understanding of the cause of death?  (complete registered cause of death, if known, below) | |  | |
| What was the mode of death? | |  | Planned palliative care |
|  | |  | Withholding, withdrawal or limitation of life-sustaining treatment |
|  | |  | Brainstem death |
|  | |  | Failed Cardiopulmonary resuscitation |
|  | |  | Witnessed event |
|  | |  | Found dead |
|  | |  | Not known |
| Expected  Unexpected | |  |  |
| Has a medical certificate of the cause of death been issued? | Yes / No / Not Known | | |
| Was this death referred to the coroner? | Yes / No / Not Applicable / Not Known | | |
| Was a post-mortem examination carried out? | Yes / No / Not Applicable / Not Known    Date of PM if known    /    /  Place of PM if known | | |
| Has an inquest been held? | Yes / No / Not Applicable / Not Yet/ Not Known    Date of Inquest if known   /    / | | |
| Registered cause of death if known (for children over 28 days) | **Ia**  **Ib**  **Ic**  **II** | | |
| Registered cause of death if known (for neonatal deaths) | 1. main diseases or conditions in infant      1. other diseases or conditions in infant      1. main maternal diseases or conditions affecting infant      1. other maternal diseases or conditions   affecting infant     1. other relevant conditions | | |

**All – please complete**

| Where was the child at the time of the event or condition which led to the death? |  | Acute Hospital |  | | Emergency Department  Paediatric Ward  Neonatal Unit  Paediatric Intensive Care Unit  Adult Intensive Care Unit  Other |
| --- | --- | --- | --- | --- | --- |
|  |  | Home of normal residence | | | |
|  |  | Other private residence | | | |
|  |  | Foster Home | | | |
|  |  | Residential Care | | | |
|  |  | Public place | | | |
|  |  | School | | | |
|  |  | Hospice | | | |
|  |  | Mental health inpatient unit | | | |
|  |  | Abroad | | | |
|  |  | Other (specify) | |  | |
|  |  | Not known | | | |

| Where was the child when the death was confirmed? |  | Acute Hospital |  | | Emergency Department  Paediatric Ward  Neonatal Unit  Paediatric Intensive Care Unit  Adult Intensive Care Unit  Other |
| --- | --- | --- | --- | --- | --- |
|  |  | Home of normal residence | | | |
|  |  | Other private residence | | | |
|  |  | Foster Home | | | |
|  |  | Residential Care | | | |
|  |  | Public place | | | |
|  |  | School | | | |
|  |  | Hospice | | | |
|  |  | Mental health inpatient unit | | | |
|  |  | Abroad | | | |
|  |  | Other (specify) | |  | |
|  |  | Not known | | | |

| **Were any of the following events known to have occurred?** | | |
| --- | --- | --- |
|  | Neonatal Death | Complete B2 - Please complete form B2 before continuing to complete the rest of this form, as you may not be required to provide any further information through Form B. |
|  | Death of a child with a life limiting condition (to be completed by the lead clinician or designated member of the palliative care team) | Complete B3 |
|  | Sudden unexpected death in infancy (to be completed by the SUDI paediatrician or designated deputy, and will almost always be completed at or immediately after the local case review meeting. In those rare instances in which there is no local case review meeting the SUDI paediatrician or designated deputy should complete this form at the conclusion of the investigation) | Complete B4 |
|  | Road traffic accident/collision | Complete B5 |
|  | Drowning | Complete B6 |
|  | Fire/burns | Complete B7 |
|  | Poisoning | Complete B8 |
|  | Other non-intentional injury/accidents/trauma | Complete B9 |
|  | Substance misuse | Complete B10 |
|  | Apparent homicide | Complete B11 |
|  | Apparent suicide | Complete B12 |

| **Circumstances of Death:**  Please provide a narrative account of the circumstances leading to the death. This should include a chronology of significant events (e.g. contact with service; changes in family circumstances) in the background history, and details of any important issues identified. **Consider**: Events leading to the death; Early family history; Pregnancy and birth; Infancy; Pre-school; School years; Adolescence |
| --- |

**C: The Child**

*This section provides information about the child and any known conditions or factors intrinsic to the child that may have contributed to the death. Please complete any information which you hold on the case.*

| Birth weight (gm or oz / Ib) | | gms        Ibs      oz | | Gestational age at birth (completed weeks) | | | |  | | |
| --- | --- | --- | --- | --- | --- | --- | --- | --- | --- | --- |
| Last known weight (gm or oz / Ib)  Date | | gms        Ibs      oz       /     / | | Last known height (ft/in or cm)  Date | | | | cm        ft       in       /     / | | |
| Any known medical conditions at the time of death?  If yes, please provide details below | | | | | | | Yes / No / Not known | | | |
| Was the child fully immunised? | | | | | | | Yes / No / Not known    Date of last immunisation    /    / | | | |
| Any known developmental impairment or disability at the time of death?  If yes, please provide details below | | | | | | | Yes / No / Not known | | | |
| Any medication at the time of death?  If yes, please provide details below | | | | | | | Yes / No / Not known | | | |
| Education/Occupation | | | | |  | Not yet in education | | | | |
|  | | | | |  | Nursery | | | | |
|  | | | | |  | School | | | | |
|  | | | | |  | College | | | | |
|  | | | | |  | Not in education | | | | |
|  | | | | |  | Left education | | |  | Employed |
|  | | | | | | | | |  | Unemployed |
| If employed, please provide occupation | | | | |  | | | | | |
| Ethnic group |  | | White | | English/Welsh/Scottish/Northern Irish/British  Irish  Gypsy or Irish Traveller  Any other White background  (please specify) | | | | | |
|  |  | | Mixed/  multiple ethnic  groups | | White and Black Caribbean  White and Black African  White and Asian  Any other mixed/multiple ethnic background (please specify) | | | | | |
|  |  | | Asian or Asian British | | Indian  Pakistani  Bangladeshi  Chinese  Any other Asian background  (please specify) | | | | | |
|  |  | | Black/  African/  Caribbean/Black British | | African  Caribbean  Any other Black/African/Caribbean background (please specify) | | | | | |
|  |  | | Other ethnic group | | Arab  Any other ethnic group (please specify) | | | | | |
|  |  | | Not known/ not stated | | | | | | | |
| Religion (please state) |  | | | | | | | | | |

| **Factors in the child:**  Please provide a narrative description of any relevant factors within the child that have not already been covered. Include any known health needs; factors influencing health; growth parameters development/educational issues; behavioural issues; social relationships; identity and independence; any identified factors in the child that may have contributed to the death. Include strengths, as well as difficulties. |
| --- |

**D: Family and Environment**

*This section provides details of the child’s family and close environment. Please complete with any information known to you.*

**Please circle or tick your responses**

|  | Age/DoB | Gender | Relationship to child and/or family | Occupation | Living in primary household?[[1]](#footnote-2) |
| --- | --- | --- | --- | --- | --- |
| Mother |  | F | Mother |  | Y / N / NK |
| Father |  | M | Father |  | Y / N / NK |
| Other significant others *(e.g. Mother’s partner; significant carer. Please number and complete any information known; further adults can be added below)* | | | | | |
| 1 |  |  |  |  | Y / N / NK |
| 2 |  |  |  |  | Y / N / NK |
| 3 |  |  |  |  | Y / N / NK |
| 4 |  |  |  |  | Y / N / NK |
| Siblings (*Please number and complete any information known; further siblings can be added below, please include step and half siblings)* | | | | | |
| 1 |  |  |  |  | Y / N / NK |
| 2 |  |  |  |  | Y / N / NK |
| 3 |  |  |  |  | Y / N / NK |
| 4 |  |  |  |  | Y / N / NK |
| 6 |  |  |  |  | Y / N / NK |
| 7 |  |  |  |  | Y / N / NK |

| Was the child/family an asylum seeker Yes / No / Not known |
| --- |

**Further family information**

*(In relation to the primary household or other household where the child spends a significant amount of time)*

**Please circle or tick your responses**

|  | Mother | Father | Other adult 1 | Other adult 2 |
| --- | --- | --- | --- | --- |
| Smoker | Y / N / NK | Y / N / NK | Y / N / NK | Y / N / NK |
| Any Known: |  |  |  |  |
| Disability, including learning disability? | Y / N / NK | Y / N / NK | Y / N / NK | Y / N / NK |
| Physical health issues? | Y / N / NK | Y / N / NK | Y / N / NK | Y / N / NK |
| Mental health  issues? | Y / N / NK | Y / N / NK | Y / N / NK | Y / N / NK |
| Substance misuse? | Y / N / NK | Y / N / NK | Y / N / NK | Y / N / NK |
| Alcohol misuse? | Y / N / NK | Y / N / NK | Y / N / NK | Y / N / NK |
| Known to police | Y / N / NK | Y / N / NK | Y / N / NK | Y / N / NK |

| Are mother and father related to each other (excluding marriage) | Yes |  | No |  | Please provide details. |
| --- | --- | --- | --- | --- | --- |

| Any known domestic violence in the household? (please provide details below)  Yes / No / Not known |
| --- |

| **Factors in the family and environment:**  Please provide a description of any relevant factors known to you that have not been covered elsewhere.  **Consider**: family structure and functioning; wider family relationships; housing; employment and income; social integration and support; community resources. Include strengths and difficulties |
| --- |

**E: Parenting Capacity**

*The purpose of this section is to understand factors in relation to the care of the child that may have been of relevance in any way to the child’s death, and also factors that may have contributed to support and nurture of the child. Please complete any information known to you.*

| Where was the child living at the time of their death or the event leading to their death? |  | Parental home  Other relatives  Foster carers  Private fostering  Residential unit  Long stay hospital  Hospice  Other |
| --- | --- | --- |
| Who was directly looking after the child at the time of their death or the event that led to their death? (please tick all that apply) |  | Mother  Father |
|  |  | Other adults (please list and give adults relationships to the child) |
|  |  | Child/young person (please list and give age and relationships to the child) |
|  |  | Health care staff  Others (please list below) |

| Was the child subject to a child protection plan? |  | At the time of death  Previously  Not at all |
| --- | --- | --- |
| Category of most recent child protection plan: |  | Physical abuse  Neglect  Emotional abuse  Sexual abuse  Not known |
| Was the child subject to any statutory orders? |  | At the time of death  Previously  Not at all |
| Category of most recent  statutory order: |  | Police Powers of Protection  Emergency Protection Order  Interim Care Order  Care Order  Supervision Order  Residence Order  Section 20 (Children Act 1989)  Antisocial behaviour order  Other court order, please specify: |
| Had the child been assessed as a child in need under section 17 of the Children Act 1989? |  | At the time of death  Previously  Not at all |
| Were any siblings subject to a child protection plan? |  | At the time of death  Previously  Not at all |
| Were any siblings subject to any statutory orders? |  | At the time of death  Previously  Not at all |

| **Factors in the parenting capacity:**  Provide a narrative description of the parenting capacity with any relevant factors known to you and not already covered elsewhere.  **Consider** issues around provision of basic care; health care (including antenatal care where relevant); safety; emotional warmth; stimulation; guidance and boundaries; stability. Include strengths as well as difficulties. |
| --- |

**F: Service Provision**

*The purpose of this section is to obtain a profile of the services being offered to the child and family; the effectiveness of those services in supporting the child and family; and to identify any unmet needs or gaps in services. Please complete any information you are able to on your agency.*

**Details of agency involvement**

Please indicate whether any of the services listed were involved with the child, or in neonatal deaths, with the mother. Where any service was involved, please provide details in the narrative section below.

**Please circle or tick your responses**

| Agency / professional | Involved at time of death or in relation to the final illness[[2]](#footnote-3) | Involved previously |
| --- | --- | --- |
| Primary Health Care | Y / N / NK /NA | Y / N / NK /NA |
| Secondary / Tertiary Hospital Services | Y / N / NK /NA | Y / N / NK /NA |
| Secondary / Tertiary Community Health Services | Y / N / NK /NA | Y / N / NK /NA |
| Hospice Services | Y / N / NK /NA | Y / N / NK /NA |
| Child & Adolescent Mental Health | Y / N / NK /NA | Y / N / NK /NA |
| Police | Y / N / NK /NA | Y / N / NK /NA |
| Local Authority Children’s Services | Y / N / NK /NA | Y / N / NK /NA |
| Education | Y / N / NK /NA | Y / N / NK /NA |
| Connexions | Y / N / NK /NA | Y / N / NK /NA |
| Probation | Y / N / NK /NA | Y / N / NK /NA |
| Other (please specify) | Y / N / NK /NA | Y / N / NK /NA |

| If no professionals involved at the time of death, what was the last known contact of a professional from your agency? | Professional  Date of last known contact    /    /  Nature of contact  No known contact from this agency  Not known |
| --- | --- |

| Were there any identified unmet needs / gaps in services? (if yes, please provide details below) | Y / N / NK /NA |
| --- | --- |
| Were there any identified difficulties in family engagement with services? (if yes, please provide details below) | Y / N / NK /NA |

**Factors in relation to service provision**

| Please complete any information known to you in relation to service provision that has not been covered elsewhere.  **Consider** any identified services both required and provided; the nature and timing of any services provided; any gaps between child’s or family member’s needs and service provision; any issues in relation to service provision or uptake, positive/negative in relation to bereavement care. |
| --- |

| Was there a formal Critical Incident investigation – if yes, please state which specific agency | Y / N / NK /NA |
| --- | --- |

| Any other internal agency investigation (please specify) |
| --- |

| Is this child death the subject of a serious case review | Y / N / NK /NA |
| --- | --- |

| **Issues for discussion**  Include any action or learning you consider should be taken forward as a result of the child’s death; issues that require broader multi-agency discussion |
| --- |

1. If the child is living in more than one household, for example where the parents have separated, the primary household is where the child spends most of his/her time; please provide any relevant details in the narrative section. [↑](#footnote-ref-2)
2. Include all those providing services at the time of death or in relation to the final illness, even if not present at the time of the death; e.g. child on school roll; planned out patient follow up; active social work case; palliative care. [↑](#footnote-ref-3)
