# Supplementary material for: Implementation of Child Death Review in the Netherlands: results of a pilot study
Source: BMC Health Serv Res. 2016 Jul 8;16:235. doi: 10.1186/s12913-016-1500-9 (PMC4938929; doi:10.1186/s12913-016-1500-9)
Supplement: Supplementary file 2 [file 12913_2016_1500_MOESM2_ESM.odt]

Analysis Proforma

This proforma is used by the Child Death Overview Panel (CDOP) to:

- evaluate information about the child’s death;

- identify lessons to be learnt; and

- to inform an understanding of all child deaths at a national level.

Where prior to the CDOP meeting, a local case discussion is held, the local team may complete a draft Form C to be forwarded to the CDOP to inform their deliberations.

| Agencies represented at the meeting: Primary Health Care Paediatrics Hospital Services Mental Health Services Ambulance Services Police Children’s Social Care Services Schools Other (Specify) | Yes   No Yes   No Yes   No Yes   No Yes   No Yes   No Yes   No Yes   No |
| --- | --- |

| List of documents available for discussion |
| --- |
|  |

| Cause of death as presently understood |
| --- |
|  |

| Case Summary |
| --- |
| A few paragraphs at most: a summary of the background and a factual description of events leading up to death.  This should be as short as possible. |
|  |

# The CDOP should analyse any relevant environmental, extrinsic, medical or personal factors that may have contributed to the child’s death under the following headings.

For each of the four domains below, determine different levels of influence (0-3) for any identified factors:

0	-	Information not available

1	-	No factors identified or factors identified but are unlikely to have contributed to the death

2	-	Factors identified that may have contributed to vulnerability, ill-health or death

3	-	Factors identified that provide a complete and sufficient explanation for the death

This information should inform the learning of lessons at a local level. 

| Domain - Child’s needs |
| --- |
| Factors intrinsic to the child Include any known health needs; factors influencing health; development/ educational issues; behavioural issues; social relationships; identity and independence; abuse of drugs or alcohol; note strengths and difficulties |
| Please enter relevant information |
| Please tick the following boxes if these factors were present or may have contributed to the death Condition: |  | Relevance (0-3) |
| Acute / Sudden onset illness Specify: | Yes / No / NK |  |
| Chronic long term illness |
| Asthma | Yes / No / NK |  |
| Epilepsy | Yes / No / NK |  |
| Diabetes | Yes / No / NK |  |
| Other chronic illness Specify: | Yes / No / NK |  |
| Disability or impairment |
| Learning disabilities Specify: | Yes / No / NK |  |
| Motor impairment Specify: | Yes / No / NK |  |
| Sensory impairment Specify: | Yes / No / NK |  |
| Other disability or impairment Specify: | Yes / No / NK |  |
|  |
| Emotional / behavioural / mental health condition in the child Specify: | Yes / No / NK |  |
| Allergies Specify: | Yes / No / NK |  |
| Alcohol/substance misuse by the child Specify: | Yes / No / NK |  |

| Domain - family and environment |
| --- |
| Factors in the family and environment Include family structure and functioning; including parental abuse of drugs or alcohol; wider family relationships; housing; employment and income; social integration and support; community resources; note strengths and difficulties |
| Please enter relevant information |
| Please tick the following boxes if these factors were present or may have contributed to the death Condition: |  | Relevance (0-3) |
| Emotional/behavioural/mental health condition in a parent or carer Specify: | Yes / No / NK |  |
| Alcohol/substance misuse by a parent/carer Specify | Yes / No / NK |  |
| Smoking by the parent/carer in household Specify: | Yes / No / NK |  |
| Smoking by the mother during pregnancy Specify: | Yes / No / NK |  |
| Housing Specify: | Yes / No / NK |  |
| Domestic violence Specify: | Yes / No / NK |  |
| Co-sleeping Specify: | Yes / No / NK |  |
| Bullying Specify: | Yes / No / NK |  |
| Gang/knife crime Specify: | Yes / No / NK |  |
| Pets/animal assault Specify: | Yes / No / NK |  |
| Consanguinity Specify: | Yes / No / NK |  |

| Domain - parenting capacity |
| --- |
| Factors in the parenting capacity Include issues around provision of basic care; health care (including antenatal care where relevant); safety; emotional warmth; stimulation; guidance and boundaries; stability; note strengths and difficulties |
| Please enter relevant information |
| Please tick the following boxes if these factors were present or may have contributed to the death Condition: |  | Relevance (0-3) |
| Poor parenting/supervision Specify: | Yes / No / NK |  |
| Child abuse/neglect Specify: | Yes / No / NK |  |

| Domain - service provision |
| --- |
| Factors in relation to service provision  Include any identified services (either required or provided); any gaps between child’s or family member’s needs and service provision; any issues in relation to service provision or uptake |
| Please enter relevant information |
| Please tick the following boxes if these factors were present or may have contributed to the death Condition: |  | Relevance (0-3) |
| Access to health care Specify: | Yes / No / NK |  |
| Prior medical intervention Specify: | Yes / No / NK |  |
| Prior surgical intervention Specify: | Yes / No / NK |  |

The CDOP should categorise the likely/cause of death using the following schema.

This classification is hierarchical: where more than one category could reasonably be applied, the highest up the list should be marked.

| Category | Name & description of category | Tick box below |
| --- | --- | --- |
| 1 | Deliberately inflicted injury, abuse or neglect This includes suffocation, shaking injury, knifing, shooting, poisoning & other means of probable or definite homicide; also deaths from war, terrorism or other mass violence; includes severe neglect leading to death. |  |
| 2 | Suicide or deliberate self-inflicted harm  This includes hanging, shooting, self-poisoning with paracetamol, death by self-asphyxia, from solvent inhalation, alcohol or drug abuse, or other form of self-harm.  It will usually apply to adolescents rather than younger children. |  |
| 3 | Trauma and other external factors  This includes isolated head injury, other or multiple trauma, burn injury, drowning, unintentional self-poisoning in pre-school children, anaphylaxis & other extrinsic factors.  Excludes Deliberately inflected injury, abuse or neglect. (category 1). |  |
| 4 | Malignancy Solid tumours, leukaemias & lymphomas, and malignant proliferative conditions such as histiocytosis, even if the final event leading to death was infection, haemorrhage etc. |  |
| 5 | Acute medical or surgical condition  For example, Kawasaki disease, acute nephritis, intestinal volvulus, diabetic ketoacidosis, acute asthma, intussusception, appendicitis; sudden unexpected deaths with epilepsy. |  |
| 6 | Chronic medical condition  For example, Crohn’s disease, liver disease, immune deficiencies, even if the final event leading to death was infection, haemorrhage etc. Includes cerebral palsy with clear post-perinatal cause. |  |
| 7 | Chromosomal, genetic and congenital anomalies  Trisomies, other chromosomal disorders, single gene defects, neurodegenerative disease,cystic fibrosis, and other congenital anomalies including cardiac. |  |
| 8 | Perinatal/neonatal event  Death ultimately related to perinatal events, eg sequelae of prematurity, antepartum and intrapartum anoxia, bronchopulmonary dysplasia, post-haemorrhagic hydrocephalus, irrespective of age at death.  It includes cerebral palsy without evidence of cause, and includes congenital or early-onset bacterial infection (onset in the first postnatal week). |  |
| 9 | Infection  Any primary infection (ie, not a complication of one of the above categories), arising after the first postnatal week, or after discharge of a preterm baby.  This would include septicaemia, pneumonia, meningitis, HIV infection etc. |  |
| 10 | Sudden unexpected, unexplained death Where the pathological diagnosis is either ‘SIDS’ or ‘unascertained’, at any age.  Excludes Sudden Unexpected Death in Epilepsy (category 5). |  |

The panel should categorise the ‘preventability’ of the death – tick one box.

Preventable child deaths are defined in paragraphs 7.23 and 7.24 of Working Together to Safeguard Children

| Modifiable factors identified | The panel have identified one or more factors, in any domain, which may have contributed to the death of the child and which, by means of locally or nationally achievable interventions, could be modified to reduce the risk of future child deaths |  |
| --- | --- | --- |
| No Modifiable factors identified | The panel have not identified any potentially modifiable factors in relation to this death |  |
|  | Inadequate information upon which to make a judgement. NB this category should be used very rarely indeed. |  |

| Issues identified in the review |
| --- |
| List the issues identified by the review group.  This list may include the absence of certain key persons from the discussion or the lack of key documents. |
|  |

| Learning Points |
| --- |
| List the learning points that emerge.  These may well overlap with the issues and with recommendations. |
|  |

| Recommendations |
| --- |
| List any recommendations, even if already picked up as learning points or ‘issues’ |
| Specific agency |
| LSCB |
| Regional |
| National |

| Follow up plans for the family, where relevant |
| --- |
|  |

| Possible Actions |
| --- |
| Should this death be referred to another agency or Authority (e.g. Police, Coroner, Health and Safety Executive, Serious Case Review panel) for further investigation or enquiry? If so, please state |
|  | Yes |  | No |  | Already done |
| If yes please specify; |  |
